# Carbon dots derived from *Sanguisorbae Radix* mitigate intestinal injury after severe burns: mechanisms involving barrier enhancement and oxidative stress amelioration

**DOI:** 10.3389/fmolb.2026.1796404

**Published:** 2026-06-04

**Authors:** Liwei Liu, Yusheng Zhao, Baigong Feng, Meizhuo Li, Shengbo Xu, Xingxiu Yan, Junkang Wang, Duo Li, Chuanan Shen

**Affiliations:** 1 Medical School of Chinese PLA, Beijing, China; 2 Senior Department of Burns and Plastic Surgery, the Fourth Medical Center of Chinese PLA General Hospital, Beijing, China

**Keywords:** burn, carbon dots, intestinal injury, oxidative stress, Sanguisorbae radix

## Abstract

**Background:**

As of now, effective clinical treatments for burn-induced intestinal injury are still limited. Carbon dots (CDs), recognized for their unique biological activities, present a promising avenue for the development of innovative nanomedicine-based therapies aimed at addressing intestinal barrier dysfunction following burns.

**Methods:**

This study reports the successful synthesis of a novel class of CDs—specifically derived from *Sanguisorbae Radix carbonisata* (SRC-CDs)—through a green, one-step pyrolysis method, using *Sanguisorbae Radix* as the sole precursor. A comprehensive suite of analytical techniques, including high-resolution transmission electron microscopy (HR-TEM), dynamic light scattering (DLS), fluorescence spectroscopy, and Fourier-transform infrared (FTIR) spectroscopy, was employed to meticulously characterize the physicochemical properties, particle morphology, surface chemistry, and optical features of the synthesized SRC-CDs. Furthermore, the intestinal protective efficacy of SRC-CDs fabricated at varying pyrolysis temperatures (260 °C, 310 °C, 360 °C, and 410 °C) was evaluated in a murine model of 30% total body surface area (TBSA) full-thickness scald injury. Assessments included intestinal permeability biomarkers, tight junction protein expression, inflammatory cytokine levels, and oxidative stress parameters.

**Results:**

Notably, SRC-CDs synthesized across a spectrum of pyrolysis temperatures (260 °C, 310 °C, 360 °C, and 410 °C) consistently exhibited particle diameters within a defined nanoscale range (2.0–8.0 nm) and featured a rich surface chemistry characterized by diverse functional groups, irrespective of the specific thermal conditions employed. Importantly, *in vivo* administration of SRC-CDs conferred significant protection against burn-induced intestinal injury. This protective effect was manifested through the substantial mitigation of histopathological damage and the functional restoration of the intestinal barrier. Mechanistically, SRC-CDs treatment effectively restored intestinal barrier integrity, as demonstrated by a marked reduction in circulating levels of permeability markers, intestinal fatty acid binding protein (I-FABP) and diamine oxidase (DAO), coupled with a robust upregulation of key tight junction proteins, ZO-1 and occludin, within the intestinal mucosa. At a molecular level, SRC-CDs exerted pronounced anti-inflammatory and antioxidant actions, significantly suppressing the levels of pro-inflammatory cytokines (TNF-α, IL-1β, and IL-6) and the lipid peroxidation product malondialdehyde (MDA), while concurrently enhancing the activities of the endogenous antioxidant enzymes superoxide dismutase (SOD) and glutathione (GSH).

**Conclusion:**

This study developed biocompatible SRC-CDs via green pyrolysis, demonstrating their efficacy in mitigating burn-induced intestinal injury. SRC-CDs enhanced barrier integrity, reduced inflammation and oxidative stress, with the 360 °C-synthesized variant showing optimal bioactivity, thereby supporting the traditional use of SR and proposing a novel nanomaterial strategy for managing critical burn complications.

## Introduction

Massive fluid depletion subsequent to severe burns commonly induces hypovolemic shock, characterized by inadequate perfusion of critical organs (Burgess et al., 2022). This state renders the intestine highly prone to ischemia-hypoperfusion injury (Zhou et al., 2022). The pathophysiological sequelae include impaired energy homeostasis in intestinal epithelial cells, an exacerbated inflammatory response with copious cytokine release, and direct cellular injury culminating in necrosis and apoptosis (McMahan et al., 2022). The collective deterioration of epithelial structure and function undermines the gut barrier, thereby facilitating systemic influx of intestinal microbiota and endotoxins (Luck et al., 2021). This translocation is a key driver of enteric endotoxemia, with the potential to progress to sepsis and multiple organ dysfunction syndrome.

A central consequence is the progressive loss of tight junction integrity (Arumugam et al., 2025). The structural and functional integrity of the intestinal epithelium is critically dependent on the precise expression, post-translational modification, and supramolecular organization of tight junction complexes. Key transmembrane scaffold proteins such as zonula occludens-1 (ZO-1) and occludin are essential for the formation and maintenance of the paracellular seal (Zhang Z. et al., 2025; Tang et al., 2025). The combined insult of mucosal hypoperfusion, ischemia-reperfusion pathology, and maladaptive inflammatory signaling following thermal injury induces substantial degradation of these tight junction complexes (Luck et al., 2022). The resultant increase in paracellular permeability facilitates systemic translocation of endotoxins and other luminal contents (Li et al., 2025). Currently, therapeutic strategies specifically targeting early intestinal hypoperfusion after severe burns are limited globally. Conventional fluid resuscitation only partially attenuates this complication, highlighting an urgent unmet need for the exploration and development of novel treatment modalities and pharmacological agents aimed at preserving intestinal barrier integrity.

Carbon dots (CDs) are a category of zero-dimensional carbon nanomaterials defined by their sub-10 nm dimensions (Deb and Chowdhury, 2024; Manikandan and Lee, 2022). Initially reported in 2004, these nanoparticles have attracted extensive attention in biomedicine for applications such as bioimaging (Das et al., 2024), drug delivery (Bartkowski et al., 2024), and oncotherapy (Lu et al., 2024), which is largely attributed to their outstanding optical performance (Shi et al., 2023), high aqueous solubility (Wu et al., 2023), low cost (Xu et al., 2024), favorable biocompatibility (Kamble et al., 2024), and eco-friendliness (Farshidfar et al., 2023). During the past 2 decades, substantial advances have been made in the development and application of CDs, with their prospects being especially notable in biomedical and pharmaceutical domains (Wang et al., 2024; Tu et al., 2023).

Chinese herbal medicine-derived CDs (CHM-CDs) represent an emerging category of nanomaterials synthesized via high-temperature pyrolysis from traditional Chinese medicinal materials or their extracts (Zhao et al., 2025; Ai et al., 2024). Typically measuring less than 10 nm in diameter, CHM-CDs share a similar preparation approach with conventional CDs and retain their advantageous physicochemical characteristics (Zhu et al., 2024; Sun et al., 2023). These properties endow CHM-CDs with considerable promise for advanced applications, including nanomedicine and tumor-targeted drug delivery systems.

*Sanguisorbae Radix Carbonisata* (SRC), processed from its precursor *Sanguisorbae Radix* (SR), demonstrates therapeutic properties in cooling blood to arrest bleeding and detoxifying to support wound healing. It is commonly applied in clinical practice for managing hemorrhagic conditions, burns, scalds, and intestinal ailments, with modern pharmacological studies having substantiated its efficacy. In earlier research, our group innovatively took the processing methodology of SRC as a focal point, merging concepts and approaches from materials science and traditional Chinese medicine. Through the application of advanced nanoscale characterization techniques alongside methodical pharmacological assays, we were the first to identify that bioactive nanoscale CHM-CDs constitute the principal material basis for the pharmacological activity of SRC.

This study presents the first report on a green synthesis approach for SRC-CDs using a one-step pyrolysis process, entirely without chemical reagents. The intestinal protective efficacy of SRC-CDs synthesized at varying temperatures (260 °C, 310 °C, 360 °C, and 410 °C) was evaluated in a mouse model of full-thickness scald involving 30% total body surface area (TBSA). Systematic assessment of intestinal permeability markers, tight junction proteins, inflammatory cytokines, and oxidative stress indicators further clarified the mechanisms underlying the mitigation of post-burn intestinal injury by SRC-CDs, providing enhanced theoretical grounding for their potential medical application.

## Materials and methods

### Materials

All chemical reagents and kits were commercially sourced. Specifically, *SR* was acquired from Beijing Qiancao Herbal Pieces Co., Ltd. (Beijing, China, 24103013). Dialysis membranes (MWCO: 1000 Da, HF132640) were obtained from Beijing Ruida Henghui Technology Development Co., Ltd (Beijing, China). ELISA kits for intestinal fatty acid binding protein (iFABP, MM-0044M1), tumor necrosis factor-α (TNF-α, MM-0132M1), interleukin-1β(IL-1β, MM-0040M1), and interleukin-6 (IL-6, MM-0163M1) measurements, as well as assay kits for diamine oxidase (DAO, MM-0389M1), Superoxide Dismutase (SOD, MM-0389M1), Glutathione (GSH, MM-0661M1), and malondialdehyde (MDA, MM-0897M1), were purchased from Jiangsu Meimian Industrial Co., Ltd. (Jiangsu, China). Antibodies against Zonula Occludens-1 (ZO-1, 13663) and occludin (91131) were sourced from Cell Signaling Technology (USA), Inc., while the DAPI fluorescent quencher containing 4′, 6-diamidino-2-phenylindole (28718–90–3) was procured from Beijing Solaibao Technology Co., Ltd. (Beijing, China).

### Animals

This study was performed in compliance with Chinese national regulations on experimental animals and in full adherence to the National Guidelines for the Care and Use of Laboratory Animals. The experimental protocol received prior review and approval from the Institutional Animal Care and Use Committee of Chinese PLA Medical School. Forty-eight male C57BL/6J mice (supplied by SiPeiFu Biotechnology Co., Ltd., Beijing, China) were housed in an SPF-grade facility under controlled environmental conditions (temperature: 24 °C ± 1 °C; humidity: 50% ± 10%; 12-h light/dark cycle). Food and water were available *ad libitum*.

### Preparation of SRC-CDs

SRC-CDs were synthesized via a one-step high-temperature pyrolysis method using SR as the precursor. Briefly, 240 g of SR was placed in crucibles, sealed with foil and lids to create an airtight environment, and then calcined in a muffle furnace (TL0612, Beijing Zhong Ke Ao bo Innovation Co., Ltd., China) at 260 °C, 310 °C, 360 °C and 410 °C for 1 h to obtain SRC. After cooling to room temperature, the SRC was ground into a fine powder using a micro pulverizer. The homogeneous black residue was then subjected to two cycles of extraction with boiling water (100 °C, 1 h each). The combined extract was filtered through a 0.22 μm cellulose acetate membrane and concentrated. Finally, SRC-CDs were purified by dialysis against deionized water (MWCO: 1000 Da) for 7 days.

### Characterization of SRC-CDs

Transmission electron microscopy (TEM; Tecnai G220, FEI Company, USA) operating at an accelerating voltage of 100 kV was used to examine the microstructure, particle size distribution, and morphology of SRC-CDs. Atomic-scale lattice fringes and crystalline structural features were studied by high-resolution transmission electron microscopy (HRTEM; JEN-1230, Japan Electron Optics Laboratory, Japan). The optical characteristics of SRC-CDs were evaluated by ultraviolet-visible (UV-vis) absorption spectroscopy (CECIL, Cambridge, UK) and fluorescence (FL) spectroscopy (F-4500, Tokyo, Japan) to record absorption spectra and photoluminescence behavior, respectively. Additionally, Fourier transform infrared spectroscopy (FTIR; Thermo Fisher, Fremont, CA, USA) was utilized to identify surface functional groups. Colloidal stability and particle size distribution were evaluated via zeta potential measurements and dynamic light scattering (DLS) performed on a Zetasizer Nano ZS90 system (Malvern Instruments, UK).

### Models of a 30% TBSA full-thickness scald in mice and drug treatment

Fifty-six mice (8–10 weeks, 22–26 g) were randomly allocated into seven experimental groups, with eight animals in each. The groups included: a sham group (Sham), a no-rehydration group (NR, intraperitoneal administration), a rehydration group (AR, intraperitoneal administration), and four treatment groups that received SRC-CDs synthesized at different temperatures (260 °C, 310 °C, 360 °C, and 410 °C; all administered intraperitoneally at a dose of 4 mg/kg). The volume of deionized water or SRC-CDs solution administered intraperitoneally was 0.2 mL per mouse (20 g body weight), equivalent to 10 mL/kg body weight. This volume is within the recommended limits for intraperitoneal injection in mice (maximum 20 mL/kg for bolus injection) as established by standard laboratory animal guidelines. The dose of SRC-CDs (4 mg/kg) was prepared by dissolving the carbon dots in an appropriate volume of deionized water to achieve this administration volume. Prior to inducing injury, all mice were anesthetized with isoflurane, using 5% for induction and 2% for maintenance. Following adequate anesthesia, the dorsal fur was shaved from a standardized region covering approximately 30% of the total body surface area (TBSA), defined as the skin area between the bilateral hindlimbs and ears. Except for the Sham group, all animals received a full-thickness scald injury by immersing the exposed area in 90 °C water for 10 s (Huang et al., 2019). Immediately post-burn, all animals in the burn groups received buprenorphine (0.05 mg/kg, subcutaneous) for postoperative analgesia. Sham group mice underwent the same immersion procedure using 37 °C water to account for procedural effects without causing thermal damage. No interventions were administered post-procedure to the Sham controls. Treatment groups received intraperitoneal injections of SRC-CDs, while the rehydration control group received an equal volume of deionized water via the same route.

### Sample collection

At 12 h post-injury, all animals were anesthetized and blood samples (1.5–2.5 mL) were obtained from the orbital venous plexus. The collected blood was centrifuged at 3,000 rpm for 15 min to separate serum, which was then aliquoted and stored at −80 °C until further analysis. Additionally, an 8–10 cm segment of the proximal ileum was harvested and thoroughly rinsed with ice-cold physiological saline. A 1 cm portion of the tissue was immersion-fixed in 4% paraformaldehyde. The remaining ileal segment was opened longitudinally, and the mucosal layer was gently scraped off using glass slides, rapidly frozen in liquid nitrogen, and maintained at −80 °C for subsequent assays.

### Histopathological evaluation

Following fixation in 4% paraformaldehyde, intestinal samples were embedded in paraffin and sectioned at 5 μm thickness. Histopathological changes were evaluated using hematoxylin and eosin (HE) staining and microscopic observation, with representative images systematically captured for documentation. Lesion severity was quantitatively assessed according to the Chiu’s scoring system, which employs a six-grade scale: grade 0, intact mucosa; grade 1, subepithelial spaces at villous tips; grade 2, partial separation of epithelium from lamina propria; grade 3, focal epithelial detachment along villi; grade 4, complete villous denudation; and grade 5, severe mucosal destruction with hemorrhage or ulceration. This standardized scoring method facilitates objective evaluation of intestinal barrier injury progression.

### Assessment of relevant biochemical indicators

Serum levels of intestinal permeability biomarkers, specifically iFABP and DAO, were determined via commercially available enzyme-linked immunosorbent assay (ELISA) kits in strict accordance with the manufacturer’s established procedures.

### Immunofluorescence staining

Immunofluorescence staining was conducted to analyze the expression and subcellular distribution of the tight junction proteins ZO-1 and occludin in intestinal tissue sections. After air-drying at room temperature, sections were heat-fixed at 65 °C for 15 min and deparaffinized using sequential xylene and graded ethanol washes (100%, 95%, and 70%). Antigen retrieval was performed in citrate buffer at 65 °C. Endogenous peroxidase activity was blocked with 3% H_2_O_2_, followed by permeabilization with 0.2% Triton X-100 for 15 min. Non-specific binding was suppressed by blocking with 5% BSA for 2 h. Sections were then incubated overnight at 4 °C with primary antibodies against ZO-1 and occludin (1:200 dilution). After washing, species-matched fluorescent secondary antibodies (Alexa Fluor 594 and 488) were applied for 2 h. Fluorescence microscopy (Nikon Eclipse Ti) was used to examine protein localization and relative expression levels under consistent imaging conditions.

### The expression of inflammatory cytokines in the intestinal mucosal tissue

The frozen intestinal mucosal tissues, stored at −80 °C, were homogenized on ice in cold PBS with a tissue grinder (120 Hz, 2 min, 4 °C). After centrifugation at 12,000 rpm for 15 min (4 °C), the supernatant was collected and analyzed for TNF-α, IL-1β, and IL-6 levels using commercial ELISA kits according to the manufacturer’s protocols.

### The levels of oxidative stress in the intestinal mucosal tissue

Following centrifugation, the supernatant of intestinal mucosal homogenates was collected and used for the quantification of MDA, SOD, and GSH levels with commercial assay kits, as per the manufacturer’s instructions.

### Statistical analysis

Statistical analyses were conducted using IBM SPSS Statistics (version 20.0). Differences among multiple groups were evaluated by one-way analysis of variance (ANOVA), followed by Fisher’s least significant difference (LSD) post-hoc test for intergroup comparisons. Quantitative data are expressed as mean ± standard deviation (SD).

## Results

### Morphological characteristics and crystalline structure of SRC-CDs

**FIGURE 1:**
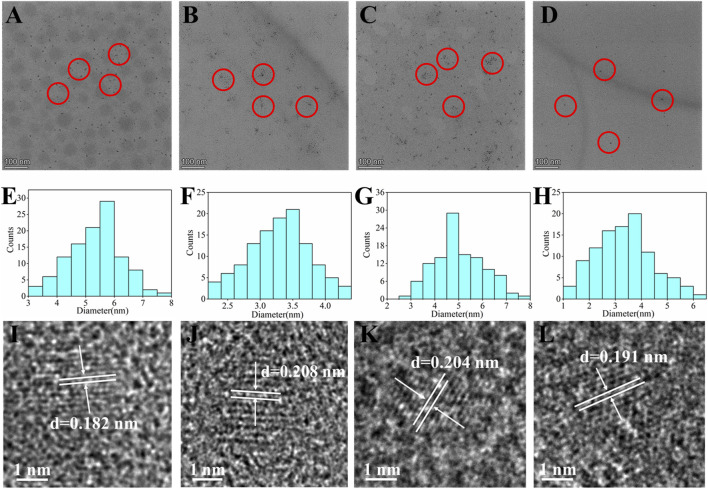
Transmission electron microscope (TEM) images of SRC-CDs prepared with different temperature conditions. Low resolution transmission electron micrograph: **(A)** 260 °C; **(B)** 310 °C; **(C)** 360 °C; **(D)** 410 °C, SRC-CDs are indicated by red circles. Particle size distribution chart: **(E)** 260 °C; **(F)** 310 °C; **(G)** 360 °C; **(H)** 410 °C. High resolution transmission electron micrograph: **(I)** 260 °C; **(J)** 310 °C; **(K)** 360 °C; **(L)** 410 °C.

**FIGURE 2:**
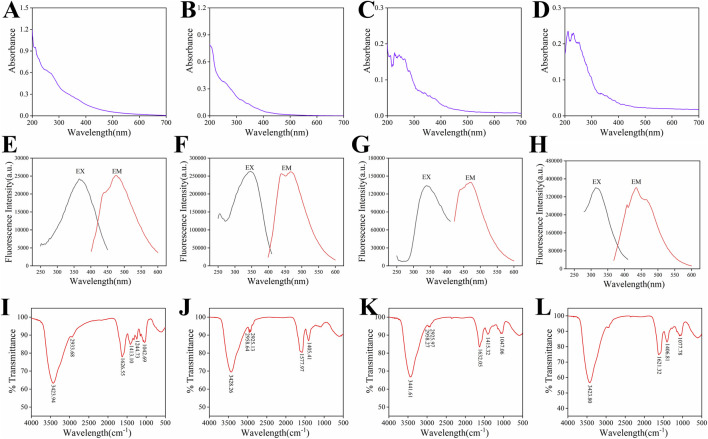
Spectra of SRC-CDs prepared with different temperature conditions. Ultraviolet-visible spectrum: **(A)** 260 °C; **(B)** 310 °C; **(C)** 360 °C; **(D)** 410 °C. Fluorescence spectrum: **(E)** 260 °C; **(F)** 310 °C; **(G)** 360 °C; **(H)** 410 °C. Fourier transform infra-red spectrum: **(I)** 260 °C; **(J)** 310 °C; **(K)** 360 °C; **(L)** 410 °C.

The morphology, dimensions, and size distribution of SRC-CDs synthesized with different reaction durations were analyzed using transmission electron microscopy (TEM). High-resolution TEM (HR-TEM) was further applied to evaluate their crystalline structure via lattice spacing measurements. Representative results are compiled in Figure 1. Low-resolution TEM images indicated that all SRC-CDs adopted nearly spherical shapes with Gaussian-type size distributions. Specifically, the SRC-CDs fabricated at 260 °C (Figure 1A) possess larger dimensions. In comparison, those synthesized at 310 °C (Figure 1B) are notably smaller. The sample produced at 360 °C (Figure 1C) demonstrates optimal size uniformity. Conversely, the material obtained at 410 °C (Figure 1D) shows pronounced size polydispersity. As summarized in Figures 2E–H, the size distribution ranges of each sample are as follows: the sample prepared at 260 °C spans 3.0–8.0 nm, the SRC-CDs synthesized at 310 °C range from 2.0 to 4.5 nm, those obtained at 360 °C measure between 2.5 and 8.0 nm, and the product prepared at 410 °C covers 1.0–6.5 nm. Corresponding HR-TEM analysis revealed lattice spacings of 0.182 nm (Figure 1I), 0.208 nm (Figure 1J), 0.204 nm (Figure 1K), and 0.191 nm (Figure 1L). Well-defined particle boundaries were discernible in all samples, confirming the distinct crystalline nature of the synthesized SRC-CDs.

### UV-vis absorption and fluorescence properties of SRC-CDs

**FIGURE 3:**
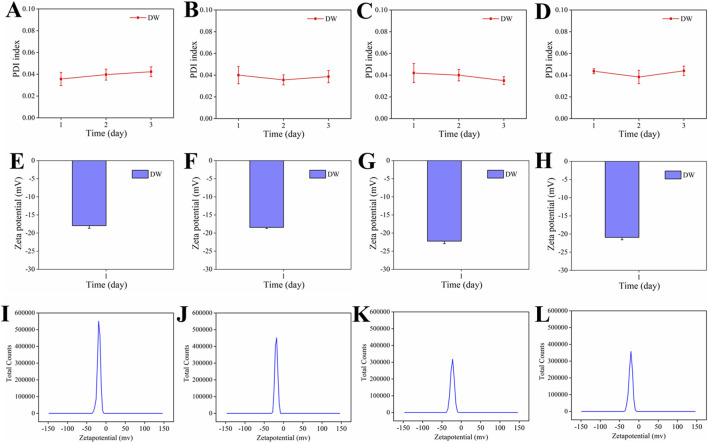
Dispersion stability and surface charge characterization of SRC-CDs prepared with different temperature conditions. Dynamic light scattering analysis: **(A)** 260 °C; **(B)** 310 °C; **(C)** 360 °C; **(D)** 410 °C. Zeta potential analysis: **(E)** 260 °C; **(F)** 310 °C; **(G)** 360 °C; **(H)** 410 °C. Surface charge characterization: **(I)** 260 °C; **(J)** 310 °C; **(K)** 360 °C; **(L)** 410 °C.

The UV-Vis absorption spectra of the synthesized SRC-CDs are presented in Figure 3. All samples prepared at 260 °C (Figure 2A), 310 °C (Figure 2B), 360 °C (Figure 2C), and 410 °C (Figure 2D) displayed an absorption peak in the 260–280 nm region, along with a broad shoulder centered around 350 nm.

The fluorescence properties of the SRC-CDs were characterized by FL spectroscopy. As shown in Figures 2E–H, the maximum excitation and emission wavelengths exhibited a clear dependence on synthesis temperature. Specifically, for samples prepared at 260 °C, 310 °C, 360 °C, and 410 °C, the optimal excitation wavelengths were 366, 344, 339, and 314 nm, respectively, while their corresponding maximum emission wavelengths were 475, 465, 468, and 435 nm. This systematic spectral shift is likely attributable to variations in particle size and the distribution of surface functional groups arising from the different reaction temperatures.

### Surface chemical composition of SRC-CDs

The surface functional groups of the SRC-CDs were analyzed by FTIR spectroscopy, as shown in Figures 2I–L. The spectra revealed characteristic absorption peaks that varied with synthesis temperature. The sample prepared at 260 °C (Figure 2I) displayed peaks at 3423 cm^-1^, 2933 cm^-1^, 1626 cm^-1^, 1413 cm^-1^, and 1042 cm^-1^. For the 310 °C sample (Figure 2J), notable absorptions were observed at 3428 cm^-1^, 2958 cm^-1^, 2925 cm^-1^, 1577 cm^-1^, and 1405 cm^-1^. The SRC-CDs obtained at 360 °C (Figure 2K) showed bands at 3441 cm^-1^, 2958 cm^-1^, 2925 cm^-1^, 1632 cm^-1^, 1415 cm^-1^, and 1047 cm^-1^, while the 410 °C product (Figure 2L) exhibited peaks at 3423 cm^-1^, 1621 cm^-1^, 1406 cm^-1^, and 1077 cm^-1^. The broad absorption band around 3430 cm^-1^ can be assigned to O–H or N–H stretching vibrations (Garcia-Millan et al., 2023). The signals near 2920 cm^-1^ correspond to C–H stretching of–CH_2_– groups (Najmalden Ghaibullah Ghaibullah et al., 2024). The peak at approximately 1630 cm^-1^ indicates the presence of C=O bonds, and the absorption around 1400 cm^-1^ likely arises from C–N stretching vibrations (Kasi et al., 2025). The band near 1050 cm^-1^ is consistent with C–O–C stretching modes (Marković et al., 2023). These results confirm that SRC-CDs synthesized at different temperatures possess abundant surface functional groups, including hydroxyl, amino, and carboxyl moieties, which enhance their water solubility and hydrophilicity.

### Stability of SRC-CDs

**FIGURE 4:**
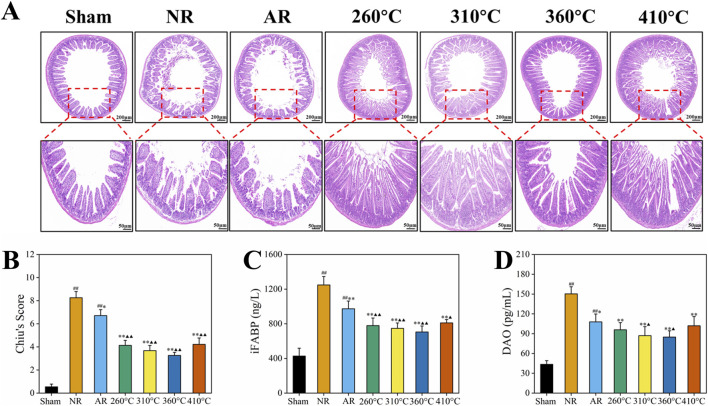
Effects of SRC-CDs synthesized with different temperature conditions (260 °C, 310 °C, 360 °C and 410 °C) on intestinal histopathology and serum markers of gut barrier permeability in severely burned mice. **(A)** Histopathological sections of intestine tissue (Scale bar: ×400). **(B)** Statistical analysis of the pathological histological scores. Serum concentrations of **(C)** iFABP and **(D)** DAO. Analysis of mice treated with sham group (Sham), no rehydration group (NR), rehydration group (AR), groups treated with SRC-CDs prepared at 260 °C (4 mg/kg, i.p.), 310 °C (4 mg/kg, i.p.), 360 °C (4 mg/kg, i.p.), and 410 °C (4 mg/kg, i.p.). ^##^ *P* < 0.01 vs. sham group; **P < 0.01, *P < 0.05 vs. NR group; ^▲▲^ *P* < 0.01, ^▲^ *P* < 0.05 vs. AR group.

The dispersion stability of the SRC-CDs in deionized water (DW) was assessed by DLS over a period of 3 days. As shown in Figures 3A–4D, the PDI values remained stable throughout this timeframe for SRC-CDs synthesized at reaction durations of 260 °C (0.0357 ± 0.0060, 0.0397 ± 0.0050, 0.0423 ± 0.0045), 310 °C (0.0400 ± 0.0079, 0.0357 ± 0.0046, 0.0387 ± 0.0057), 360 °C (0.0420 ± 0.0089, 0.0400 ± 0.0053, 0.0350 ± 0.0036), and 410 °C (0.0437 ± 0.0023, 0.0383 ± 0.0061, 0.0440 ± 0.0044). These findings indicate that the SRC-CDs maintain excellent dispersion stability regardless of their reaction time.

The surface charges of the SRC-CDs were characterized by zeta potential analysis. As shown in Figures 4E–L, all samples exhibited negative zeta potentials, measured at −17.93 ± 0.75 mV (260 °C), −18.43 ± 0.31 mV (310 °C), −22.23 ± 0.68 mV (360 °C), and −20.93 ± 0.64 mV (410 °C). The net negative surface charge is likely due to the presence and ionization of oxygen-containing functional groups on the SRC-CD surfaces. Moreover, this inherent negative charge contributes significantly to the long-term colloidal stability of the SRC-CDs in aqueous solutions.

### SRC-CDs alleviate intestinal injury in severely burned mice

Histological examination at 12 h post-injury (Figure 4A) revealed marked pathological alterations in intestinal tissues across groups. The sham group displayed normal intestinal architecture, characterized by well-aligned villi, intact cellular structure, and absence of inflammation or hemorrhage. In contrast, the NR group exhibited the most severe injury, manifesting as extensive submucosal hemorrhage, pronounced inflammatory infiltration, villous disruption with patchy epithelial sloughing, and partial degradation of the lamina propria. Treatment with SRC-CDs synthesized at different temperatures (260 °C, 310 °C, 360 °C, and 410 °C) alleviated submucosal hemorrhage and inflammation while preserving villous integrity to varying degrees. Among these, the group treated with SRC-CDs synthesized at 360 °C showed the most pronounced therapeutic effect.

The severity of intestinal barrier damage was assessed using the Chiu’s scoring system, as illustrated in Figure 4B. Compared to the Sham group, the NR (non-rehydration) group exhibited a significantly elevated Chiu’s score (*P* < 0.01), confirming substantial intestinal injury. Post-injury intervention with different treatments markedly mitigated this damage. Both the AR (rehydration only) group and the groups receiving SRC-CDs synthesized at 260 °C, 310 °C, 360 °C, and 410 °C showed significantly lower Chiu’s scores relative to the NR group (*P* < 0.01). Notably, treatment with SRC-CDs, regardless of synthesis temperature, resulted in scores that were significantly lower than those in the AR group (*P* < 0.01), underscoring the superior therapeutic efficacy of SRC-CDs over rehydration alone. Furthermore, among the SRC-CD-treated groups, the 360 °C-synthesized SRC-CDs demonstrated the most pronounced protective effect, achieving the lowest Chiu’s score, which indicates optimal restoration of intestinal barrier integrity.

Serum concentrations of the intestinal barrier biomarkers iFABP and DAO progressively increased in the NR group compared with the Sham group (Figures 4C,D), demonstrating a deterioration of intestinal permeability in the absence of resuscitation. Quantitative analysis confirmed that serum iFABP and DAO concentrations were significantly elevated in the NR group compared with the Sham group (*P* < 0.01). Conversely, in the AR group and across all SRC-CD treatment groups (synthesized at 260 °C, 310 °C, 360 °C, and 410 °C), these marker levels were markedly lower than those in the NR group (*P* < 0.05, *P* < 0.01). Notably, intervention with 360 °C-synthesized SRC-CDs exhibited superior efficacy, significantly lowering serum iFABP and DAO concentrations relative to the AR group (*P* < 0.05, *P* < 0.01).

### SRC-CDs restore intestinal barrier integrity by upregulating tight junction proteins

**FIGURE 5:**
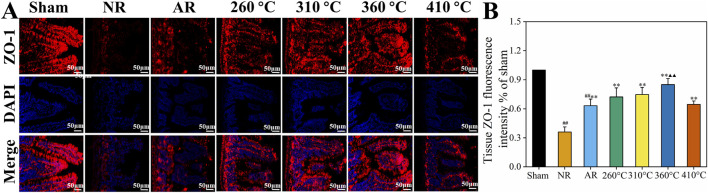
Immunofluorescence analysis of ZO-1 protein expression in intestinal tissue of severely burned mice treated with SRC-CDs synthesized with different temperature conditions (260 °C, 310 °C, 360 °C and 410 °C). **(A)** Immunofluorescence images of ZO-1 (magnification: ×400). **(B)** Quantitative analysis of ZO-1 fluorescence intensity. Analysis of mice treated with sham group (Sham), no rehydration group (NR), rehydration group (AR), groups treated with SRC-CDs prepared at 260 °C (4 mg/kg, i.p.), 310 °C (4 mg/kg, i.p.), 360 °C (4 mg/kg, i.p.), and 410 °C (4 mg/kg, i.p.). ^##^ *P* < 0.01 vs. sham group; **P < 0.01 vs. NR group; ^▲▲^ *P* < 0.01 vs. AR group.

Immunofluorescence analysis (Figures 5A,B) showed a significant downregulation of the tight junction protein ZO-1 in the intestinal epithelium of the NR group compared with the Sham group (*P* < 0.01). In contrast, ZO-1 expression was markedly increased in both the AR group and all SRC-CDs intervention groups synthesized at different temperatures (260 °C, 310 °C, 360 °C, and 410 °C) relative to the NR group (*P* < 0.01). Notably, SRC-CDs synthesized at 360 °C exhibited a stronger up-regulatory effect on ZO-1 expression compared to the AR group (*P* < 0.01). Although not statistically significant, SRC-CDs prepared at other temperatures also showed a tendency toward elevated ZO-1 expression.

**FIGURE 6:**
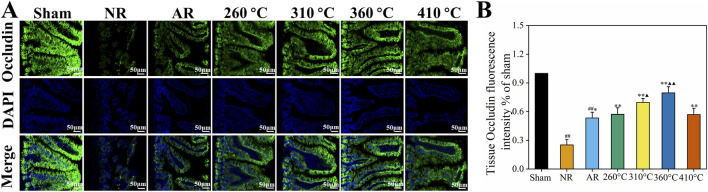
Immunofluorescence analysis of Occludin protein expression in intestinal tissue of severely burned mice treated with SRC-CDs synthesized with different temperature conditions (260 °C, 310 °C, 360 °C and 410 °C). **(A)** Immunofluorescence images of Occludin (magnification: ×400). **(B)** Quantitative analysis of Occludin fluorescence intensity. Analysis of mice treated with sham group (Sham), no rehydration group (NR), rehydration group (AR), groups treated with SRC-CDs prepared at 260 °C (4 mg/kg, i.p.), 310 °C (4 mg/kg, i.p.), 360 °C (4 mg/kg, i.p.), and 410 °C (4 mg/kg, i.p.). ^##^ *P* < 0.01 vs. sham group; **P < 0.01, *P < 0.05 vs. NR group; ^▲▲^ *P* < 0.01, ^▲^ *P* < 0.05 vs. AR group.

Immunofluorescence analysis (Figures 6A,B) demonstrated that the expression of the tight junction protein Occludin in the intestinal epithelium was significantly decreased in the NR group compared to the Sham group (*P* < 0.01). Relative to the NR group, both the AR group and all SRC-CDs intervention groups synthesized at different temperatures (260 °C, 310 °C, 360 °C, and 410 °C) exhibited significant upregulation of Occludin expression (*P* < 0.01 and *P* < 0.05). Further intergroup comparisons revealed that SRC-CDs synthesized at 310 °C and 360 °C were more effective than the AR group in promoting Occludin expression (*P* < 0.01 and *P* < 0.05), with the 360 °C-synthesized SRC-CDs showing the most potent therapeutic effect. Although not statistically significant, SRC-CDs prepared at the remaining temperatures also demonstrated an upward trend in Occludin expression.

### SRC-CDs ameliorate intestinal inflammation and oxidative stress post-severe burn

**FIGURE 7:**
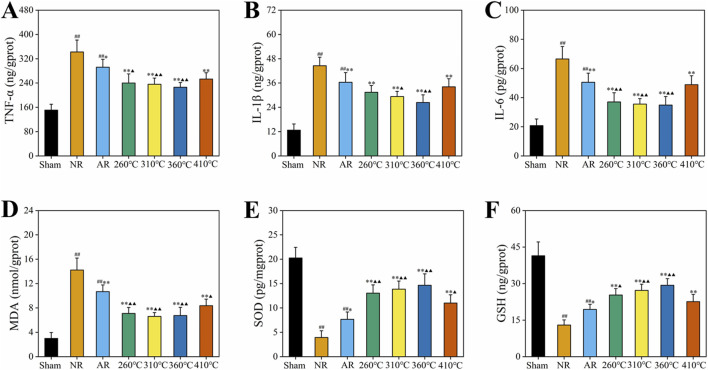
Effects of SRC-CDs synthesized with different temperature conditions (260 °C, 310 °C, 360 °C and 410 °C) on inflammatory cytokines and oxidative stress in the intestinal mucosal epithelium. **(A)** tumour necrosis factor-α (TNF-α), **(B)** interleukin (IL)-1β, **(C)** IL-6, **(D)** malondialdehyde (MDA), **(E)** superoxide dismutase (SOD), and **(F)** glutathione (GSH). Analysis of mice treated with sham group (Sham), no rehydration group (NR), rehydration group (AR), groups treated with SRC-CDs prepared at 260 °C (4 mg/kg, i.p.), 310 °C (4 mg/kg, i.p.), 360 °C (4 mg/kg, i.p.), and 410 °C (4 mg/kg, i.p.). ^##^ *P* < 0.01 vs. sham group; **P < 0.01, *P < 0.05 vs. NR group; ^▲▲^ *P* < 0.01,^▲^ *P* < 0.05 vs. AR group.

As shown in Figures 7A–C, levels of the pro-inflammatory cytokines TNF-α (342.64 ± 39.06 pg/mL), IL-1β (44.57 ± 4.20 pg/mL), and IL-6 (66.57 ± 8.55 pg/mL) in the intestinal mucosa were significantly elevated in the NR group compared with the Sham group (TNF-α: 150.88 ± 19.28; IL-1β: 12.76 ± 3.02; IL-6: 20.81 ± 4.51; *P* < 0.01). Relative to the NR group, both the AR group (TNF-α: 292.39 ± 26.14; IL-1β: 36.37 ± 4.85; IL-6: 50.51 ± 6.35) and all four SRC-CDs intervention groups-synthesized at 260 °C (TNF-α: 239.90 ± 30.14; IL-1β: 31.42 ± 3.36; IL-6: 37.05 ± 6.24), 310 °C (TNF-α: 235.77 ± 20.50; IL-1β: 29.32 ± 2.56; IL-6: 35.58 ± 3.71), 360 °C (TNF-α: 226.10 ± 15.51; IL-1β: 26.37 ± 3.80; IL-6: 34.87 ± 5.87), and 410 °C (TNF-α: 252.89 ± 21.27; IL-1β: 34.12 ± 3.98; IL-6: 48.84 ± 6.13)-showed significant reductions in these inflammatory mediators (*P* < 0.05 or *P* < 0.01). Notably, the SRC-CDs group synthesized at 360 °C exerted a more potent anti-inflammatory effect than the AR group, with significantly lower levels of TNF-α, IL-1β, and IL-6.

As shown in Figure 7D, the level of the oxidative stress marker MDA in the intestinal mucosa was significantly increased in the NR group (14.23 ± 1.97 nmol/mg prot) compared to the Sham group (3.00 ± 0.97 nmol/mg prot, *P* < 0.01). Relative to the NR group, both the AR group (10.67 ± 1.11 nmol/mg prot) and all SRC-CDs intervention groups-260 °C (7.10 ± 0.97), 310 °C (6.61 ± 0.63), 360 °C (6.76 ± 1.34), and 410 °C (8.37 ± 1.07)-displayed varying degrees of reduction in MDA levels (*P* < 0.05, *P* < 0.01). Notably, the SRC-CDs group synthesized at 360 °C achieved significantly lower MDA levels than the AR group.

In parallel, the expression levels of the antioxidant markers SOD (Figure 7E) and GSH (Figure 7F) in the intestinal mucosa were markedly downregulated in the NR group. (SOD: 3.94 ± 1.38 U/mg prot; GSH: 12.99 ± 2.11 μmol/g prot, *P* < 0.01) compared to the Sham group (SOD: 20.26 ± 2.19 U/mg prot; GSH: 41.43 ± 5.66 μmol/g prot, *P* < 0.01). Compared to the NR group, both the AR group (SOD: 7.66 ± 1.51 U/mg prot; GSH: 19.40 ± 2.12 μmol/g prot, *P* < 0.05) and all SRC-CDs intervention groups-260 °C (SOD: 13.05 ± 1.69, GSH: 25.32 ± 2.62), 310 °C (SOD: 13.86 ± 1.66, GSH: 27.21 ± 2.56), 360 °C (SOD: 14.66 ± 2.34, GSH: 29.30 ± 2.72), and 410 °C (SOD: 11.01 ± 1.70, GSH: 22.61 ± 2.96)-showed significant upregulation of SOD and GSH levels (*P* < 0.05, *P* < 0.01). Among these, the SRC-CDs synthesized at 360 °C demonstrated a more potent effect than the AR group in elevating both SOD and GSH levels, indicating the strongest therapeutic efficacy.

## Discussion

Nanotechnology has significantly propelled medical innovation, with nanomedicines greatly enhancing the therapeutic potential of traditional agents. Despite this promise, the widespread application of many new nanotherapeutics is constrained by challenges related to stability, safety, manufacturing complexity, and cost. Amidst these limitations, CHM-CDs present a compelling alternative. Characterized by facile synthesis, abundant precursors, and robust safety and stability profiles, these bioactive nanoparticles are regarded as a fundamental component underlying the efficacy of carbonized herbal medicines (Qiang et al., 2023; Zhou et al., 2024).

CHM-CDs represent a class of zero-dimensional carbon nanomaterials, generally under 10 nm in diameter, that are synthesized from natural Chinese medicinal herbs or their extracts (Zhang J. et al., 2023; Liu et al., 2024). Beyond preserving the bioactivity inherent to their herbal precursors, CHM-CDs also possess the favorable physicochemical characteristics typical of conventional carbon dots-including high solubility, tunable size distribution, good biocompatibility, and modifiable surface functional groups-which collectively enhance their suitability for biomedical applications (Zhang J. et al., 2025; Xiao et al., 2025). Unlike conventional inorganic materials such as metals, salts, or ceramics, CHM-CDs originate from medicinal herbs with well-documented therapeutic value (Zhou et al., 2025; Zhang et al., 2026). This unique source confers not only the structural benefits of nanomaterials but also intrinsic bioactivity and pharmacological functions. As a result, CHM-CDs show promising potential across a spectrum of advanced biomedical fields, such as drug delivery, tumor therapy, bioimaging, anti-inflammatory and antioxidant interventions, and tissue regeneration (Etefa and Dejene, 2025; Singh et al., 2024). Their development thus offers an innovative platform bridging traditional Chinese medicine modernization and contemporary nanomedicine.

Relative to conventional Chinese herbal medicine, CHM-CDs are characterized by an ultra-small particle size (<10 nm), low toxicity, high biocompatibility, favorable dispersibility and stability, along with a diverse array of surface functional groups (Paveethra et al., 2024). These advantageous physicochemical attributes underpin their improved therapeutic performance. As an illustration, functional CDs synthesized from *Magnetite* and *Medicated Leaven* have been shown to markedly alleviate intestinal bleeding, suppress colonic inflammation, repair colonic barrier damage, and further modulate gut microbiota and the inflammatory microenvironment in ulcerative colitis (Wu et al., 2024). Correspondingly, CDs derived from *Coptidis Rhizoma* enhance intestinal tight junction protein expression in murine models of ulcerative colitis, significantly reduce inflammatory cell infiltration, and effectively alleviate oxidative stress (Mou et al., 2023).

In this study, an innovative approach was employed to synthesize SRC-CDs derived from the SR using a straightforward and environmentally benign pyrolysis method, with SR serving as the exclusive precursor. The synthesized SRC-CDs displayed a homogeneous particle size distribution, each measuring less than 10 nm in diameter. Characterization of the SRC-CDs by UV-vis spectroscopy revealed consistent spectral profiles across all synthesis temperatures, each featuring a distinct absorption peak within the 260–280 nm region accompanied by a broad shoulder centered near 350 nm. The former absorption band is attributable to the π-π* electronic transitions of aromatic carbon domains within the carbon dot structure, indicating the presence of conjugated sp^2^ hybridized (Wang et al., 2023). The latter, weaker and broader band is typically associated with n-π* transitions originating from unsaturated functional groups containing heteroatoms, such as C=O or C–N bonds, on the surface of the SRC-CDs (Raypah et al., 2025). These spectral features confirm the successful carbonization of the SR precursor and the preservation of functional groups critical for their bioactivity. Notably, the position and intensity of these absorption bands showed minimal variation across synthesis temperatures (260 °C–410 °C), suggesting that the fundamental carbon core structure and surface chemistry conferring optical absorption are established even at relatively low pyrolysis temperatures.

However, the subtle differences in the fluorescence emission maxima (ranging from 435 to 475 nm) among SRC-CDs prepared at different temperatures likely reflect variations in particle size distribution and the relative abundance of specific surface functional groups, which can modulate the band gap and electronic transition energies (Romulo et al., 2025). This structure-property relationship underscores the tunability of SRC-CDs' optical characteristics through precise control of synthesis parameters, which may in turn influence their biological interactions and therapeutic efficacy (Nazari et al., 2025). Integrated characterization through FTIR and DLS verified the abundant presence of hydrophilic functional groups on the surface of SRC-CDs, endowing them with outstanding aqueous solubility and colloidal stability.

Based on these results, we proceeded to investigate the potential biomedical applications of SRC-CDs prepared at varying temperatures (260 °C, 310 °C, 360 °C, and 410 °C), placing special emphasis on elucidating their underlying protective mechanisms against post-burn intestinal injury.

Following severe burns, inflammation emerges as a central driver of intestinal pathology. This is mediated by the marked upregulation of key pro-inflammatory cytokines, such as TNF-α, IL-1β, and IL-6, within the intestinal tissue, which directly contributes to mucosal barrier disruption and epithelial damage (Al-Qahtani et al., 2024). The excessive accumulation of reactive oxygen species constitutes a central event in intestinal tissue damage following severe burns. This cascade initiates lipid peroxidation of unsaturated fatty acids within cell membranes, leading to loss of membrane integrity and function, subsequent cellular injury, and eventual exacerbation of intestinal mucosal barrier disruption (Bonetti et al., 2024). Our results demonstrate that SRC-CDs significantly attenuated pathological intestinal damage following burn injury, reduced serum levels of intestinal permeability biomarkers iFABP and DAO, and upregulated the expression of tight junction proteins ZO-1 and occludin, indicating a potent barrier-enhancing effect. Moreover, SRC-CDs effectively suppressed the expression of pro-inflammatory cytokines TNF-α, IL-1β, and IL-6 in the intestinal mucosa, decreased the oxidative stress marker MDA, and elevated the activities of the antioxidant enzymes SOD and glutathione GSH. These findings collectively reveal the multi-protective role of SRC-CDs through synergistic anti-inflammatory and antioxidant mechanisms.

This study confirms that SRC-CDs can effectively modulate oxidative stress and inflammatory responses, which may represent one of the key mechanisms underlying their ability to alleviate intestinal injury and preserve barrier integrity. Among these, the group treated with SRC-CDs prepared at 360 °C showed the most substantial reduction in all biomarkers, further highlighting the influence of synthesis parameters on treatment outcome.

The observation that SRC-CDs synthesized at 360 °C exhibited superior intestinal protective efficacy compared to those prepared at other temperatures warrants a conceptual explanation based on structure–activity relationships. We propose that this temperature-dependent bioactivity arises from an optimal convergence of physicochemical properties. At moderate pyrolysis temperatures (360 °C), the carbonization process achieves a balanced state wherein particle size distribution is sufficiently uniform for enhanced cellular uptake, while surface functional groups—particularly oxygen-containing moieties such as hydroxyl and carbonyl—are optimally preserved to confer antioxidant capacity and facilitate biological interactions. Concurrently, the carbon core attains an appropriate degree of graphitization, maintaining structural integrity while preserving edge defects that serve as active centers for radical scavenging. The relatively high negative surface charge at this temperature further enhances colloidal stability, preventing aggregation and maintaining effective surface area for tissue engagement. Lower temperatures may yield incomplete carbonization with insufficient functional group exposure and underdeveloped graphitic domains, limiting electron transfer capacity and bioactivity. Conversely, excessive temperatures may cause thermal decomposition of bioactive surface motifs and excessive graphitization with loss of defect sites, reducing therapeutic potential. This conceptual framework aligns with literature demonstrating that synthesis temperature critically influences the balance between carbon core development and functional group preservation in biomass-derived carbon dots, ultimately determining their biological performance (Mintz et al., 2019; Zhang B. et al., 2023; Sayyad et al., 2024).

This study successfully synthesized biocompatible SRC-CDs and systematically investigated their protective roles and mechanisms in severe burn-induced intestinal injury. Future research directions include: (1) elucidating the precise cellular and molecular pathways mediated by SRC-CDs; (2) assessing their long-term biosafety and pharmacokinetic profiles *in vivo*; (3) examining their therapeutic efficacy in other injury models, such as ischemia-reperfusion and sepsis; and (4) optimizing formulation strategies and advancing preclinical development to support potential clinical translation.

## Conclusion

In this work, biocompatible Sanguisorbae Radix-derived carbon dots (SRC-CDs) were synthesized through a green pyrolysis method, and their therapeutic efficacy against burn-induced secondary intestinal injury was systematically evaluated. The nanomaterials demonstrated excellent aqueous dispersibility and rich surface functional groups, effectively enhancing intestinal barrier function by reducing permeability, upregulating tight junction protein expression, and preserving mucosal integrity. Additionally, SRC-CDs exhibited synergistic anti-inflammatory and antioxidant effects, significantly mitigating key pathological pathways in post-burn intestinal damage. Pharmacodynamic studies further indicated that SRC-CDs prepared under optimized conditions (360 °C with precise temperature control) possessed the highest bioactivity. This study not only provides scientific support for the traditional use of SR, but also proposes a novel strategy for designing multi-targeted herbal-based nanomaterials, highlighting their translational potential in managing burn-related complications in critical care medicine.

## Data Availability

The raw data supporting the conclusions of this article will be made available by the authors, without undue reservation.
